# The Dynamic SUMOylation Changes and Their Potential Role in the Senescence of APOE4 Mice

**DOI:** 10.3390/biomedicines12010016

**Published:** 2023-12-20

**Authors:** Yangqi Xu, Wenwen Cai, Shaoming Sang, Xiaoqin Cheng, Boru Jin, Xiangteng Zhao, Chunjiu Zhong

**Affiliations:** 1 Department of Neurology, Zhongshan Hospital, Fudan University, Shanghai 200032, China; 2 Shanghai Key Laboratory for Tumor Microenvironment and Inflammation, Department of Biochemistry and Molecular Cell Biology, Institute of Medical Sciences, Shanghai Jiao Tong University School of Medicine, Shanghai 200025, China

**Keywords:** SUMOylation, SENP, APOE4, Alzheimer’s disease, aging

## Abstract

The ε4 allele of apolipoprotein E (APOE4) and aging are the major risk factors for Alzheimer’s disease (AD). SUMOylation is intimately linked to the development of AD and the aging process. However, the SUMOylation status in APOE4 mice has not been uncovered. In this study, we investigated SENP1 and SUMOylation changes in the brains of aged APOE3 and APOE4 mice, aiming to understand their potential impact on mitochondrial metabolism and their contribution to cellular senescence in APOE4 mice. Concurrently, SUMO1-conjugated protein levels decreased, while SUMO2/3-conjugated protein levels increased relatively with the aging of APOE4 mice. This suggests that the equilibrium between the SUMOylation and deSUMOylation processes may be associated with senescence and longevity. Our findings highlight the significant roles of SENP1 and SUMOylation changes in APOE4-driven pathology and the aging process.

## 1. Introduction

Alzheimer’s disease (AD) is the most prevalent form of dementia, pathologically characterized by abnormal protein aggregates, including misfolded amyloid β (Aβ) in amyloid plaques and hyper-phosphorylated tau protein aggregations as neurofibrillary tangles [1]. The ε4 allele of the apolipoprotein E (*APOE4*) gene is the key genetic risk factor for AD [2]. It has been well documented that APOE could interact with Aβ and promote its aggregation, influence tau neurofibrillary degeneration, and exert effects on inflammatory processes, membrane trafficking, and mitochondrial energy dysfunction [3,4,5,6]. Nevertheless, the specific contributions of each of these mechanisms to APOE4-driven pathology in AD remain unclear.

Small ubiquitin-like modifier modification (SUMOylation), a crucial post-translational protein modification, involves the covalent conjugation of a member of the SUMO family to the lysine residue of a substrate protein. This process influences the cellular localization and biological activity of target proteins in various cellular processes [7]. SUMOylation is dynamically regulated by the activity-dependent redistribution of SUMOylation machinery and is rapidly reversed by the isopeptidase activity of SUMO/sentrin-specific proteases (SENPs), which strongly influence the conjugation/deconjugation balance of SUMO-targeted proteins. Among six SENPs, most of the work on their roles in neurons has been focused on SENP1, SENP2, SENP3, and SENP6 [8,9,10]. In AD, SUMO modification affects pivotal proteins related to Aβ and tau metabolism, mitochondrial biogenesis and metabolism, and synaptic functions [11,12,13,14]. However, there is a paucity of studies exploring the SUMOylation status in APOE mice. To gain a deeper understanding of SUMOylation in AD and uncover potential regulatory mechanisms and pathways, we investigated how SUMOylation changes in aged APOE4 mice. Our observations revealed increased SENP1 expression and decreased SUMO1-ylation level in the brains of aged APOE4 mice, potentially impacting mitochondrial metabolism. Additionally, across three age stages (8, 16, and 24 months old), we noted a decline in SUMO1-conjugated protein levels and a relative increase in SUMO2/3-conjugated protein levels with the aging of APOE4 mice. These findings shed light on how the balance of SUMO/deSUMOylation may offer new insights into understanding how APOE modulates the risk of AD and senescence.

## 2. Materials and Methods

### 2.1. Experimental Animals

APOE-targeted replacement mice were originally developed on a C57Bl/6 background to express human APOE3 or APOE4 genes, in place of the mouse APOE gene, under the control of the endogenous murine promoter [15,16]. In this manuscript, mice homozygous for either the human APOE3 or human APOE4 genes are denoted as APOE3 and APOE4 mice, respectively. The humanized APOE3 and APOE4 male mice were procured from Taconic (Germantown, NY, USA) by the Model Animal Research Centre of Nanjing University and subsequently bred for this study. The genotype was confirmed by the polymerase chain reaction analysis of tail biopsies (Supplementary Materials). All animals were housed in standard specific pathogen-free (SPF) conditions with ad libitum access to sterilized water and food. Animal handling and experiment procedures were performed in accordance with the guide for the care and use of laboratory animals by the Medical Experimental Animal Administrative Committee of Fudan University. Every effort was made to reduce animal stress and to minimize animal usage. The APOE3 and APOE4 mice were sacrificed at the age of 8, 16, and 24 months old, respectively. Their body weight and blood glucose were monitored.

### 2.2. In Vivo RNAi of SENP1

The sequence used for RNAi targeting SENP1 was GACCTCAAGTGGATTATCAAA. Both shRNA for SENP1 and Control were cloned into a vector containing synapsin (Syn) promoter-driven enhanced green fluorescent protein (eGFP), subsequently packed into adeno-associated virus (AAV). AAV-loading shRNA or Control shRNA (shCtrl) was injected intracerebroventricularly as previously described [17]. Anesthetized with isoflurane, the mice were fixed on the stereotaxic apparatus for the following experiment. The skull was exposed by incising the scalp and a small burr hole was created bilaterally in the hemisphere with a dental drill at the following stereotaxic coordinates: 1.0 mm left and right of the Bregma, 0.2 mm back, and 2.5 mm deep. A volume of 5 μL virus was injected into the lateral ventricle through a needle at a constant speed for more than 10 min. The needle was held in place for 10 min and retracted slowly in case of leakage. Finally, the scalp was sutured and mice were placed on a thermostated blanket for recovery from anesthesia. Four weeks after the virus injection, the mice were sacrificed, and transcriptome analysis of frontal cortex in APOE4 mice was performed (*n* = 4).

### 2.3. Western Blot

The brain tissues were homogenized in cold NP40 lysis buffer (50 mM Tris-HCl pH 7.5, 150 mM NaCl, and 1% NP-40) supplemented with 20 mM N-ethylmaleimide (NEM, Sigma, Saint Louis, MO, USA), protease inhibitor cocktail, and phosphatase inhibitor cocktail (Thermo Scientific, Waltham, MA, USA). Protein concentrations were measured with the Pierce BCA protein assay kit according to the manufacturer’s instruction (Thermo Scientific, Waltham, MA, USA). For separation of proteins, 40 μg of protein was loaded in each well for electrophoresis on 10–12% acrylamide gels. Polyvinylidene fluoride membranes (Millipore, Burlington, MA, USA) were blocked in Tris-buffered saline with Tween 20 containing 5% nonfat dry milk for 1 h. The membrane was then probed with specific primary antibodies including SENP1 (1:1000, Abcam, Cambridge, UK), SUMO1 (1:1000, Cell Signaling Technology, Danvers, MA, USA), SUMO2/3 (1:1000, Sigma, Saint Louis, MO, USA), OXPHOS (1:1000, Abcam, Cambridge, UK), SIRT1 (1:1000, Santa Cruz, Santa Cruz, CA, USA), SIRT2 (1:1000, Abcam, Cambridge, UK), SIRT3 (1:1000, Cell Signaling Technology, Danvers, MA, USA), FOXO3A (1:1000, Cell Signaling Technology, Danvers, MA, USA), SOD2 (1:1000, Cell Signaling Technology, Danvers, MA, USA), and β-actin (1:3000, Proteintech, Chicago, IL, USA) overnight at 4 °C and then incubated for 1 h with a horseradish-peroxidase-conjugated anti-mouse IgG antibody or anti-rabbit IgG (Supplementary Table S1).

### 2.4. Immunofluorescence Staining

Mice were transcardially perfused with ice-cold PBS and fixed for 24 h in 4% PFA/PBS at 4 °C. The brains were then transferred to 30% sucrose for 2–3 days. Three randomly chosen 25-μm-thick per mouse sections were blocked in 5% BSA in PBS with 0.5% triton-X100. The sections were probed with the primary antibodies (anti-NeuN, 1:500, Abcam; anti-SENP1, 1:200, Novus biologicals, Centeennial, CO, USA) at 4 °C overnight. After washing steps, the sections were incubated for 2 h with secondary antibodies, namely Alexa 594-conjugated Donkey anti-mouse-IgG and Alexa 488-conjugated Donkey anti-rabbit-IgG (all 1:500, Invitrogen, Waltham, MA, USA), and then stained with DAPI. Immunofluorescence images were captured using Nikon A1R confocal microscope (Tokyo, Japan).

### 2.5. RNA Sequencing Data Analysis

We reanalyzed GSE95587 RNA-seq dataset from the developing human brain, utilizing the publicly available BrainSpan resource (www.brainspan.org (accessed on 7 April 2021). The dataset included fusiform gyrus tissue samples from 117 subjects, comprising 84 AD patients and 33 neurologically normal age-matched controls. The GSE95587 data had already been processed using “nRPKM” normalization for sizeFactor adjustment [18]. Additionally, RNA sequencing data of aged humanized APOE mice (GSE140205) were downloaded from the GEO database. The RNA sequencing data from the hippocampus tissue of 16-month-old APOE3 or APOE4 mice were extracted and reanalyzed (*n* = 8). Gene differential expression analysis was performed by DESeq2 software (version 1.20.0) between two different groups. DESeq2 *p* value < 0.05 |log2 Fold Change| > 0.0 was used to calculate the difference in gene expression. To infer the cellular and metabolic functions associated with the observed changes in transcript levels, the differentially expressed genes (DEGs) were categorized according to predicted protein function using Gene Ontology (GO) enrichment analysis. All DEGs were mapped to GO terms in the Gene Ontology database (http://www.geneontology.org/ (accessed on 8 November 2021). Furthermore, Gene Set Enrichment Analysis (GSEA) was performed with gene score enrichment analysis (http://www.broadinstitute.org/gsea/index.jsp (accessed on 11 November 2021).

### 2.6. Statistical Analysis

Results are presented as mean ± standard errors of the mean (SEM). Statistical analysis was performed by GraphPad Prism7. “*n*” refers to the number of animals. Comparisons between two groups were performed using two-tailed unpaired *t* test. One-way analysis of variance (ANOVA) with Tukey’s post hoc test was used to compare three or more independent groups. *p* values less than 0.05 were considered statistically significant.

## 3. Results

### 3.1. The mRNA Level of SENP1 Was Upregulated in the Brains of AD Patients and Aged APOE4 Mice

**Figure 1:**
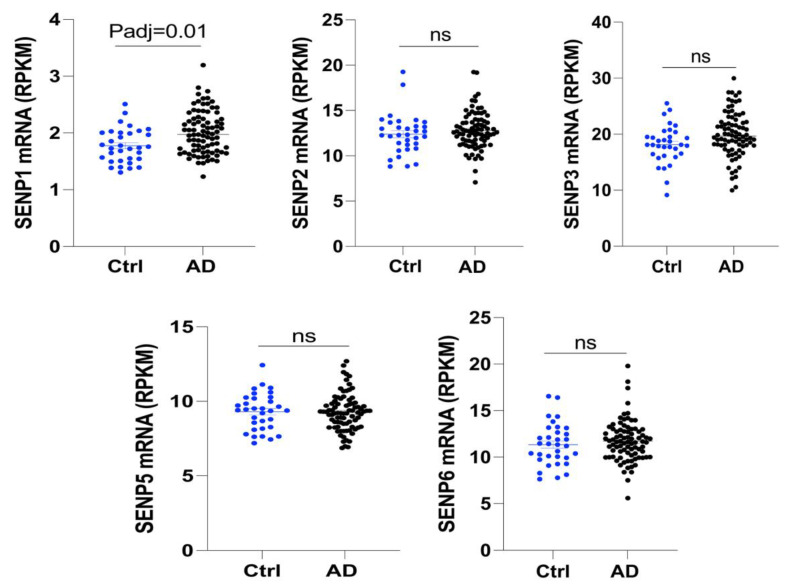
The mRNA levels of *SENPs* in the fusiform gyrus tissue of AD and normal age-matched controls. *SENP1* mRNA levels were upregulated compared to age-matched controls, while the mRNA levels of other *SENPs*, including *SENP2, SENP3, SENP5,* and *SENP6*, remained unchanged in AD patients. Data represent mean ± SEM (normal control = 33, AD patients = 84). ns, no significance. Abbreviations: AD, Alzheimer’s disease; SENPs, SUMO/sentrin-specific proteases.

**Figure 2:**
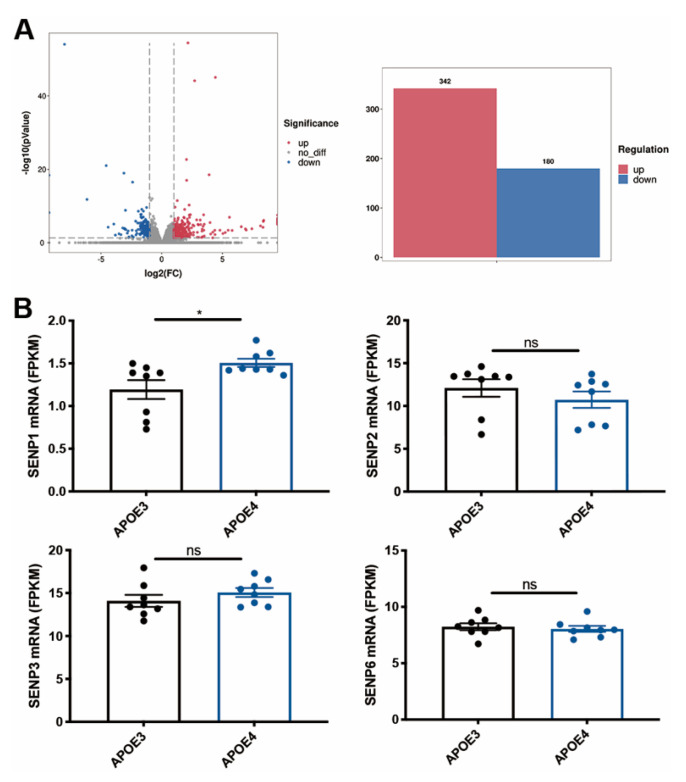
The mRNA levels of *SENPs* in aged APOE4 mice compared with age-matched APOE3 mice. (**A**) Volcano plot of DEGs in the hippocampus tissue of 16-month-old APOE4 mice compared with age-matched APOE3 mice, with a histogram showing 342 upregulated DEGs and 180 downregulated DEGs. (**B**) *SENP1* mRNA levels were upregulated, while the mRNA levels of *SENP2, SENP3*, and *SENP6* remained unchanged in 16-month-old APOE4 mice compared with age-matched APOE3 mice. Data represent mean ± SEM (*n* = 8). * *p* < 0.05. Student’s *t* test was used to determine the statistical significance of the differences. ns, no significance. Abbreviations: DEGs, differentially expressed genes.

To investigate the changes in SENPs in AD, we examined the expression levels of SENPs in the fusiform gyrus of both AD and control subjects. Our analysis revealed an upregulation in *SENP1* mRNA levels, while other *SENPs*, including *SENP2*, *SENP3*, *SENP5*, and *SENP6*, exhibited no significant changes (Figure 1). Next, we reanalyzed the RNAseq data from hippocampus tissue of 16-month-old APOE3 and APOE4 mice. Our findings identified 522 differentially expressed genes (DEGs), consisting of 342 upregulated genes and 180 downregulated genes in APOE4 mice compared to age-matched APOE3 mice (Figure 2A). Among these DEGs, *SENP1* mRNA levels were also found to be elevated in the hippocampus of aged APOE4 mice compared to age-matched APOE3 mice, while the mRNA levels of *SENP2*, *SENP3*, and *SENP6* remained unchanged (Figure 2B). These results indicate that SENP1 might play an important and unique role in the SUMOylation processes associated with APOE4-driven pathologies.

### 3.2. The SUMOylation Changes in the Brains of Aged APOE4 Mice

**Figure 3:**
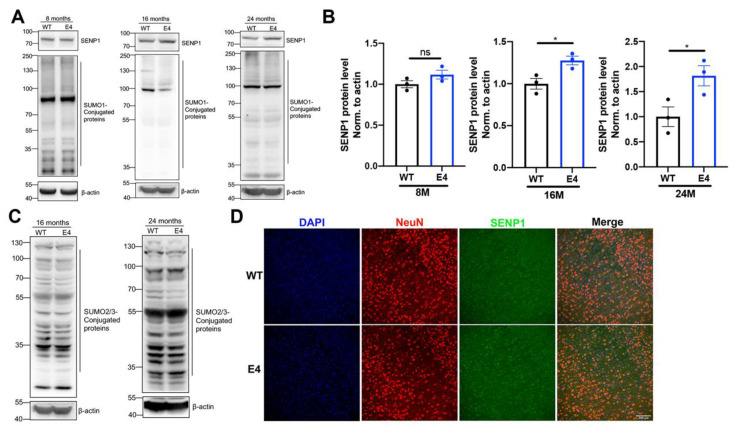
SENP1 and SUMOylation alterations in the cortical tissue between aged APOE3 and APOE4 mice. (**A**) Levels of SENP1 and SUMO1-conjugated proteins in the cortical tissue of 8-, 16-, and 24-month-old APOE3 and APOE4 mice. (**B**) Relative expression levels of SENP1 qualified and analyzed using ImageJ bundled with Java 8 (*n* = 3). (**C**) SUMO2/3-conjugated protein levels in the cortical tissue of 16-month-old and 24-month-old APOE3 and APOE4 mice. (**D**) The cortical sections of 24-month-old APOE3 and APOE4 mice stained for SENP1 (green staining) and NeuN as the neuron marker (red staining). Nuclei were stained with DAPI. Bar: 100 μm. The data are presented as the mean values ± SEMs, and significance was calculated with Student’s *t* test. * *p* < 0.05 versus the control group. ns, no significance.

Given the upregulation of *SENP1* mRNA expression in the brains of aged APOE4 mice, we further investigated the SENP1 protein expression and SUMOylation state in the brains of aged APOE4 mice. There was no change in the body weight and blood glucose between aged APOE3 and APOE4 mice (Supplementary Figure S1A). As illustrated in Figure 3A,B, SENP1 expression in the cortex of 8-month-old APOE4 mice showed no significant difference compared to age-matched APOE3 mice. However, SENP1 expression was upregulated in 16-month-old APOE4 mice, with a more pronounced increase observed in 24-month-old APOE4 mice compared to controls. Correspondingly, the levels of SUMO1-conjugated proteins, indicative of the degree of SUMO1 modification, were decreased in the cortex of aged APOE4 mice compared to age-matched APOE3 mice (Figure 3A). The levels of SUMO2/3-conjugated proteins were unchanged in the cortex of aged APOE4 mice compared to age-matched APOE3 mice (Figure 3C). Immunofluorescence staining in the cortex of 24-month-old APOE3 and APOE4 mice revealed that SENP1 was expressed abundantly in neuron (Figure 3D) and rarely in astrocytes or microglia [19].

**Figure 4:**
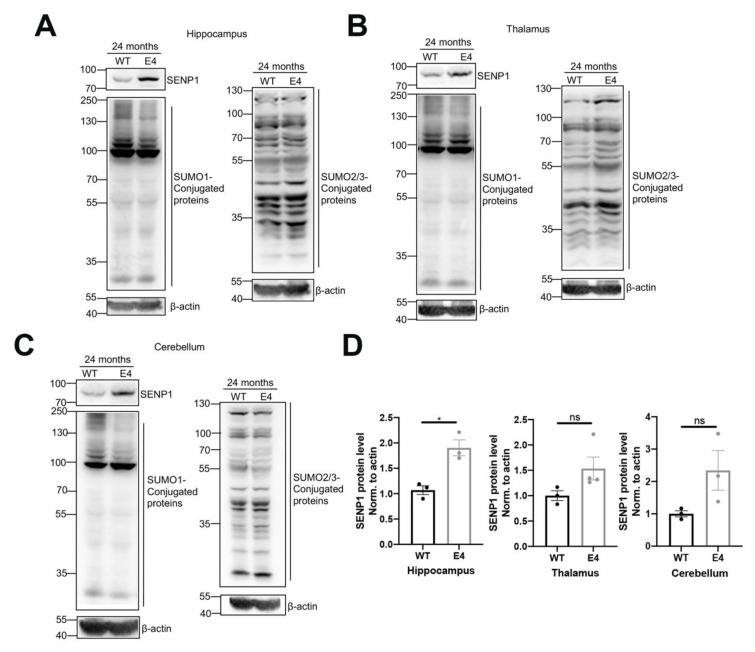
SENP1 and SUMOylation levels in the brains of aged APOE3 and APOE4 mice. SENP1 and SUMO1-conjugated protein levels in the hippocampus (**A**), thalamus (**B**), and cerebellum (**C**) of 24-month-old APOE3 and APOE4 mice (*n* = 3–4). (**D**) Relative ratio of SENP1 to the protein level of β-actin in related encephalic regions, respectively. The data are presented as the mean values ± SEMs, and significance was calculated with Student’s *t* test. * *p* < 0.05 versus the control group. ns, no significance.

Next, we examined the SUMO-conjugated protein alterations in the hippocampus, thalamus, and cerebellum of aged APOE3 and APOE4 mice. SENP1 protein expression was elevated in those encephalic regions, most prominently in the hippocampus of 24-month-old APOE4 mice compared to age-matched APOE3 mice. The amounts of SUMO1-conjugated proteins in those encephalic areas decreased, while the levels of SUMO2/3-conjugated proteins were unchanged between aged APOE3 and APOE4 mice (Figure 4). Given that SENP1 is primarily involved in the regulation of deSUMO1 modification, these results revealed that the alterations in SUMO1 modification, primarily driven by SENP1, may significantly contribute to the disordered proteins in Aβ, tau, or energy metabolisms, which leads to the development of AD.

### 3.3. The Dynamic Changes in SENP1 and SUMOylation Levels with the Aging of APOE4 Mice

**Figure 5:**
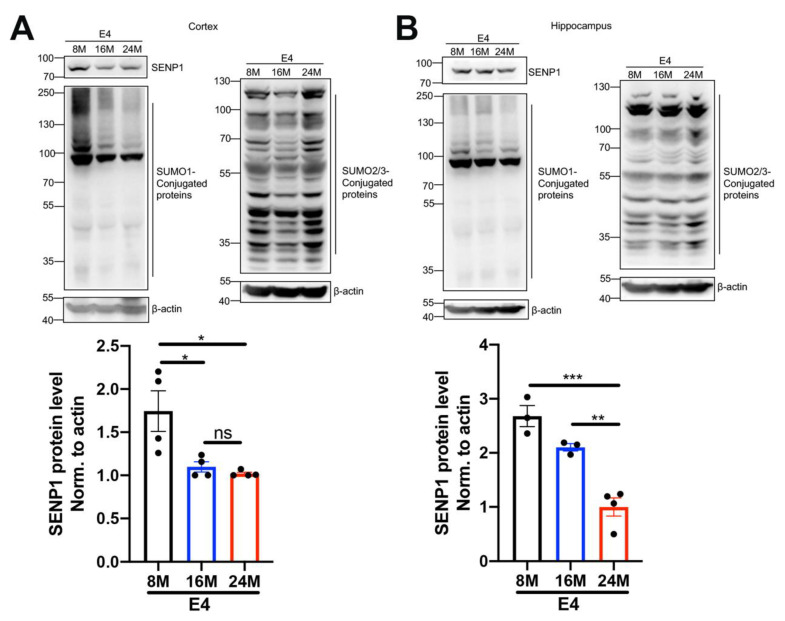
Dynamic changes in SENP1 and SUMOylation with the aging of APOE4 mice. The levels of SENP1, SUMO1-conjugated proteins, and SUMO2/3-conjugated proteins in the cortical (**A**) and hippocampal (**B**) tissue of 8-, 16- and 24-month-old APOE4 mice. The relative expression levels of SENP1 were qualified and analyzed using ImageJ bundled with Java 8 (*n* = 3–4). Data represent mean ± SEM. * *p* < 0.05, ** *p* < 0.01, *** *p* < 0.001. ns, no significance. One-way ANOVA was used to determine the statistical significance of the differences.

Aging and the APOE4 allele are major risk factors for AD [20]. Considering the association between SUMOylation and cellular senescence and aging [21], we investigated the dynamic changes in SENP1 and SUMOylation levels in APOE4 mice across different age groups. In the cortex and hippocampus of APOE4 mice, we observed that SENP1 expression and SUMO1-conjugated proteins decreased with the aging of APOE4 mice. Simultaneously, the SUMO2/3-conjugated protein levels were increased relatively in 24-month-old APOE4 mice but remained unchanged between 8- and 16-month-old APOE4 mice (Figure 5). These findings suggest that dynamic SUMOylation changes exist and possibly participate in the aging processes of APOE4 mice.

### 3.4. Mitochondrial Function Was Influenced in the Brains of Aged APOE4 Mice

**Figure 6:**
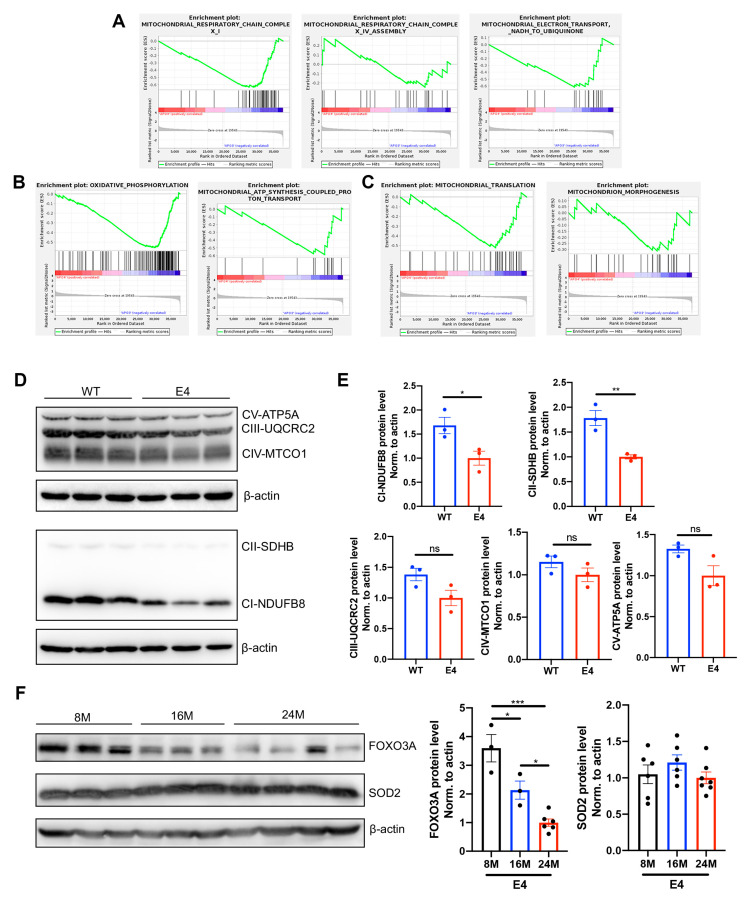
Mitochondrial function was influenced in aged APOE4 mice. GSEA enrichment plots of representative gene sets related to oxidative respiratory chain (**A**), oxidative phosphorylation (**B**), and mitochondrial translation and morphogenesis (**C**), which were downregulated in the hippocampus of 16-month-old APOE4 mice compared with age-matched APOE3 mice. (**D**) The protein levels of OXPHOS complex subunits, including CI-NDUFB8, CII-SDHB, CIII-UQCRC2, CIV-MTCO1, and CV-ATP5A, in the cortex of 24-month-old APOE3 and APOE4 mice. (**E**) Statistical results showing the normalized protein levels. The data are presented as mean values ± SEMs, and significance was calculated with Student’s *t* test (*n* = 3). (**F**) The protein expressions of FOXO3A and SOD2 in the cortex of 8-, 16-, and 24-month-old APOE4 mice. Statistical results showing the normalized protein levels. The data are presented as the mean values ± SEMs, and significance was calculated with one-way ANOVA (*n* = 3–7). * *p* < 0.05, ** *p* < 0.01, *** *p* < 0.001. ns, no significance. Abbreviations: GSEA, gene set enrichment analysis; OXPHOS, oxidative phosphorylation.

Deficits in mitochondrial function are well-known hallmarks of brain aging, prominently accentuated in neurodegenerative disorders [22]. GSEA showed that the genes enriched in oxidative respiratory chain (Figure 6A), oxidative phosphorylation (OXPHOS) (Figure 6B), mitochondrial translation, and morphogenesis (Figure 6C) were downregulated in aged APOE4 mice compared with age-matched APOE3 mice. The protein expressions of the OXPHOS system, consisting of five multi-subunit complexes (CI–CV), were decreased in the cortex of aged APOE4 mice compared with age-matched APOE3 mice (Figure 6D,E). The oxidative damage caused by mitochondria-derived reactive oxygen species is believed to be a major cause of degenerative diseases associated with aging [23]. Therefore, we further detected the oxidative stress protein superoxide dismutase 2 (SOD2) and forkhead-box protein O3a (FOXO3A) in the cortex of aged APOE4 mice. As shown in Figure 6F, FOXO3A was considerably repressed with the aging of APOE4 mice, and the SOD2 protein showed a declining trend in 24-month-old APOE4 mice.

### 3.5. SENP1 Was Involved in Mitochondrial Energy Metabolism in the Brains of Aged APOE4 Mice

**Figure 7:**
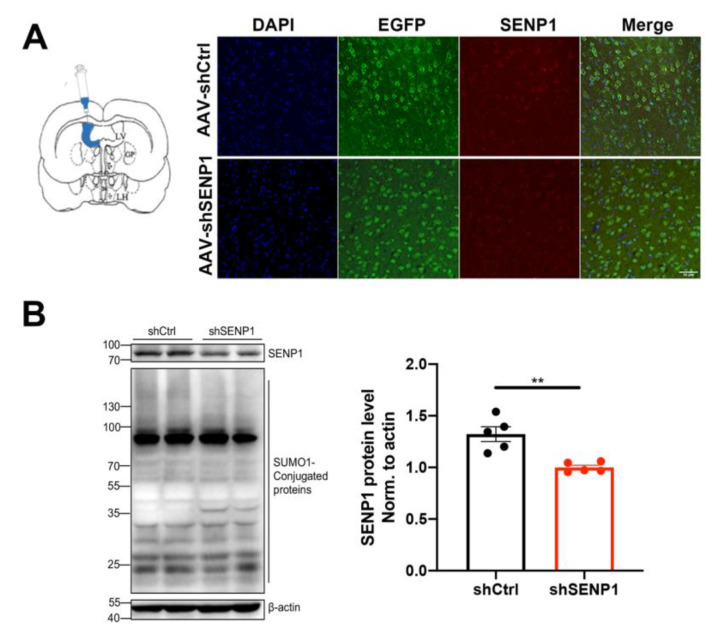
SENP1 knock-down in the brains of aged APOE4 mice. (**A**) Representative immunofluorescence images of SENP1 expression in APOE4 mouse cortical neurons (EGFP) infected by AAV loading SENP1 shRNA or Control shRNA (scale bar = 50 µm). (**B**) Efficiency of SENP1 silence by AAV and changes in SUMO1-conjugated protein levels were analyzed by WB (*n* = 5). Statistical results showing the normalized protein levels. The data are presented as the mean values ± SEMs, and significance was calculated with Student’s *t* test. ** *p* < 0.01.

**Figure 8:**
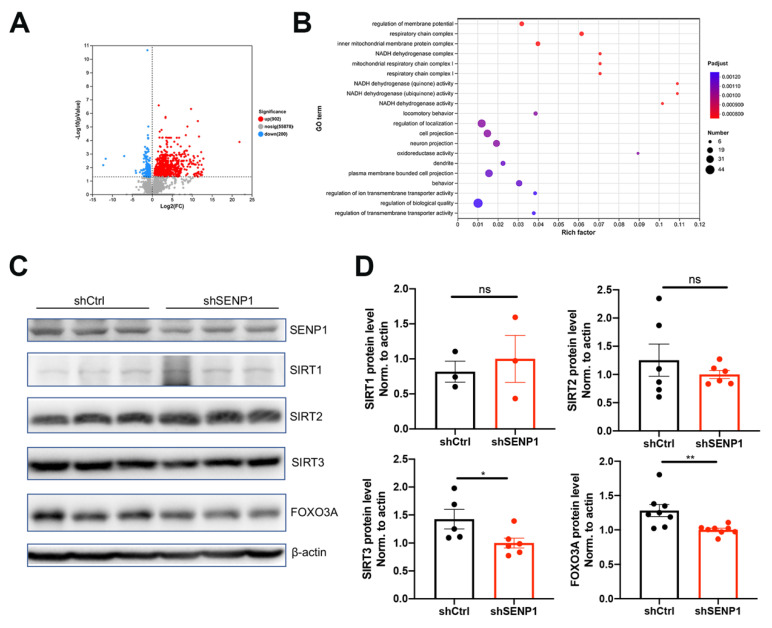
SENP1-mediated mitochondrial metabolism changes in the brains of aged APOE4 mice. (**A**) Volcano plot of DEGs in the cortex tissue between 24-month-old APOE4 mice infected with shSENP1 or shCtrl, showing that there were 902 upregulated DEGs and 200 downregulated DEGs. (**B**) The enriched pathways of downregulated DEGs by GO analysis. Rich factor is the ratio of the number of DEGs to the total number of genes in a certain pathway. The color and size of the dots represent the range of *p* value and the number of DEGs mapped to the indicated pathways, respectively. (**C**) The protein expressions of SENP1, SIRT1, SIRT2, SIRT3, and FOXO3A in the cortex of 24- month-old APOE4 mice infected with shSENP1 or shCtrl. (**D**) Statistical results showing the normalized protein levels. The data are presented as the mean values ± SEMs, and significance was calculated with Student’s *t* test (*n* = 3–8). * *p* < 0.05, ** *p* < 0.01. Abbreviations: DEGs: Differentially expressed genes; GO: Gene Ontology. ns, no significance.

APOE4 significantly impacts mitochondrial function and mitophagy, leading to alterations in oxidative stress, synapses, and cognitive function [6,24]. Furthermore, SENP1 and target deSUMOylated proteins exert their effects on mitochondrial metabolism and functions [11,25]. Thus, we speculated that SENP1 might play a role in mitochondrial metabolism and function in aged APOE4 mice. To investigate the possible role of SENP1 in aged APOE4 mice, we selectively eliminated SENP1 in the brains of aged APOE4 mice using AAV, confirming the precise deletion in neurons through immunofluorescence staining and Western blotting (Figure 7A,B). Low-molecular-weight SUMO1 conjugation showed an increasing trend by SENP1 knock-down in aged APOE4 mice. Transcriptome analysis of the cortex in aged APOE4 mice revealed 902 upregulated and 200 downregulated DEGs in APOE4 mice infected with shSENP1 compared to the control group (Figure 8A). Intriguingly, the GO enrichment analysis indicated that enriched biological processes (BPs) of downregulated DEGs were mainly centered on mitochondrial energy metabolism (Figure 8B), and BPs of upregulated DEGs were mainly centered on immune response and inflammatory process.

FOXO3A plays a role in the oxidative stress response and regulation of energy metabolism. Sirtuin 3 (SIRT3) has been shown to efficiently deacetylate FOXO3A and mediate FOXO3A to increase respiration, sustaining energy metabolism [26]. Because SENP1 is a specific SIRT3 deSUMOylation protease, we tested whether SIRT3/FOXO3A was involved in SENP1-mediated mitochondrial dysfunction in aged APOE4 mice. As depicted in Figure 8C,D, the protein levels of SIRT3 and FOXO3A were decreased in the SENP1 knock-down group compared with control group, while the levels of SIRT1 and SIRT2 remained unchanged between the two groups.

## 4. Discussion

The focus on SUMOylation, a post-translational modification (PTM), has intensified due to its implications in AD, rendering it a promising therapeutic target [8,27]. SUMO1 could directly modify β site amyloid precursor protein (APP)-cleaving enzyme 1 (BACE1) and Tau, leading to increased Aβ and tau aggregations [12]. We identified that in SENP1, a member of the SUMO-specific protease family, the mRNA level increased in the fusiform gyrus of AD patients. Nevertheless, the precise impact of SENP1 on SUMOylation status and its involvement in AD pathology remains partially understood. The mRNA or protein levels of SUMO1 were increased in several AD transgenic animal models, and SUMO-mediated alterations in specific intracellular signaling pathways contributed to AD pathologies [9,10,28]. However, in APOE4 mice, an important animal model in illustrating the pathogenesis of AD, SUMOylation status has not been delineated. In this study, we uncovered that the mRNA and protein levels of SENP1 were upregulated, and the SUMO1-conjugated protein levels decreased in the brains of aged APOE4 mice compared with age-matched APOE3 mice. These noticeable changes might participate in APOE4-triggered pathologies via influencing mitochondrial metabolism.

Two gene families, the Sirtuin and forkhead box O (FOXO) families, have been shown to play a role in the genetic regulation of longevity. The Sirtuin protein family is a class of NAD+-dependent protein deacetylases or ADP ribosyltransferases. Among Sirtuins, SIRT3 is the main mitochondrial deacetylase and plays an important role in the maintenance of mitochondrial homeostasis as a stress response protein. SIRT3 increases FOXO3A DNA-binding activity as well as FOXO3A-dependent gene expression [29], which leads to increased respiration to sustain energy metabolism, transactivates SOD and other antioxidant enzymes [30], and activates the transcription of mitophagy genes [24,31,32]. Our results revealed a significant decline of FOXO3A protein expression with the aging of APOE4 mice, implying an influence on mitochondrial oxidative stress in these mice. Furthermore, SENP1 and SUMO1-ylation levels decreased, while SUMO2/3 modification increased gradually with the aging of the APOE4 mice. The dynamic SUMOylation process in the organism underscores the importance of tight control over SUMO levels for cellular homeostasis. Nevertheless, a comprehensive understanding of the detailed effects and regulatory patterns of SENP1 and SUMOylation changes in the aging of APOE4 mice requires further exploration.

Perturbed cerebral glucose metabolism and the dysregulation of lipid metabolism are also invariant pathophysiological features of AD [33]. APOE4 is associated with decreased cerebral glucose metabolism, which occurs decades before apparent cognitive impairment in AD patients and age-matched nondemented subjects [34]. To our knowledge, APOE4 downregulates peroxisome proliferator-activated receptor-gamma coactivator-1α (PGC-1α)- SIRT3 expression and influences mitochondrial biogenesis [6,35]. Furthermore, APOE4 fragments cause mitochondrial dysfunction and neurotoxicity [36]. Our data consistently revealed downregulated mitochondrial energy metabolism in the brains of aged APOE4 mice. To ascertain whether SENP1 influences the mitochondrial function of APOE4 mice, we lowered the SENP1 protein level through AAV loading shSENP1 injection in aged APOE4 mice. Transcriptomic analysis demonstrated that downregulated DEGs were mainly enriched in pathways of mitochondrial energy metabolism following SENP1 knock-down in the brains of aged APOE4 mice. Research has shown that SENP1 deSUMOylates not only SIRT3 but also other mitochondrial proteins [25,37], promoting mitochondrial metabolism in response to metabolic stress. Additionally, a previous study reported that FOXO3A is repressed in APOE4 carriers, contributing to the dysfunction of mitophagy and mitochondrial oxidative stress. Of note, SIRT3 and FOXO3A expressions were attenuated by SENP1 deletion in aged APOE4 mice. These results suggest that SENP1 influences SIRT3-associated mitochondrial function directly or indirectly, possibly through SIRT3-FOXO3A signaling pathways in aged APOE4 mice.

A delicate balance of protein SUMOylation and deSUMOylation is an essential prerequisite for the maintenance of protein physiological functions. SUMO1 modifications participate in various biological processes including controlling the neurodevelopmental function of the transcription factor [38], participating in mitochondrial fission [39], and influencing synaptic transmission [40,41]. Conversely, the imbalance state between SUMOylation and deSUMOylation impairs synaptic function [13], promotes the inflammasome activation [42], and increases autophagic activation [43], ultimately leading to AD progression and accelerated aging [44,45]. In our study, we observed elevated SENP1 expression and decreased SUMO1-conjugated protein levels in aged APOE4 mice. However, SENP1 knock-down further downregulated mitochondrial energy metabolism pathways in aged APOE4 mice. These results imply that SENP1 elevation possibly serves as a compensatory response and protective factor in the brain of aged APOE4 mice.

## 5. Conclusions

In summary, our study elucidates the potential involvement of SENP1 in the pathologies of AD and aging driven by APOE4, with a specific impact on mitochondrial metabolism. There exist intricate mechanisms which involve the SUMOylation, dysregulation of lipid metabolism, and energy metabolism in the brains of APOE4 mice, and SENP1 could be an essential molecular target to influence the energy metabolism under the condition that lipid metabolism is disrupted. The dynamic SUMOylation changes with the aging of APOE4 mice indicate that the SUMO/deSUMOylation equilibrium relates to senescence and longevity. Our study provides hints for further exploration and unveils the possible role of SUMOylation in the brains of aged APOE4 mice.

## Data Availability

Data are available on request to the corresponding author.

## Supplementary Materials

The following supporting information can be downloaded at: https://www.mdpi.com/article/10.3390/biomedicines12010016/s1.
