# Supplementary material for: The Dynamic SUMOylation Changes and Their Potential Role in the Senescence of APOE4 Mice
Source: Biomedicines. 2023 Dec 20;12(1):16. doi: 10.3390/biomedicines12010016 (PMC10813299; doi:10.3390/biomedicines12010016)
Supplement: Supplementary file 1 [file biomedicines-12-00016-s001.zip › biomedicines-2720292-supplementary.pdf]

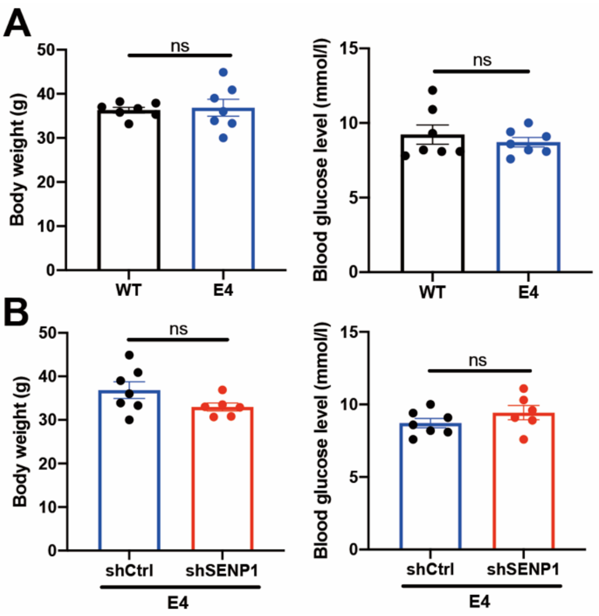

Figure S1. The body weight and blood glucose of aged APOE4 mice. A The body weight and blood glucose between 24- month-old APOE4 mice and age-matched controls. B The body weight and blood glucose between 24- month-old APOE4 mice infected with shSENP1 or shCtrl. The data are presented as the mean values ± SEMs and significance were calculated with Student's t test (n = 6-7). ns, no significance.

Supplementary methods

APOE genotype identification

DNA-containing supernatant from tail tip or toe biopsy samples was extracted by using Quick Genotyping Assay Kit for Mouse Tail (Beyotime, Cat#D7283S). The amplification conditions were 94°C for a 3-min initial strand separation, 35 cycles at 94°C for 30 s, 57°C for 30 s, 72°C for 30 s, and a 5-min final elongation step at 72°C. The primers were used to detect wild-type and mutant as follows: P1 5′- AATTTT TCCCTCCGCAGACT -3′, P2 5′- ACAGCTGCTCAGGGCTATTG -3′, and P3 5′- AGGAGGTTGAGGTGAGGATG -3′. The amplification products were separated by electrophoresis on 3% agarose gels pre-stained with ethidium bromide (Figure S2). The expected product sizes were 148 and 224bp for wild-type and mutant.

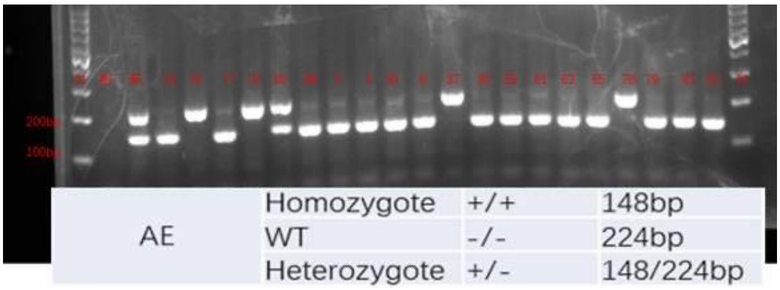

Figure S2. The APOE4 mice genotype confirmation.

Supplementary Table S1. Key resources table

REAGENT or RESOURCE	SOURCE	IDENTIFIER
Antibodies		
Rabbit monoclonal anti- SENP1	Abcam	Cat# ab108981
Rabbit polyclonal anti- SUMO1	Cell Signaling Technology	Cat# 4930
Rat monoclonal anti- SUMO2/3	Sigma	Cat# SAB4200190
Total OXPHOS rodent WB antibody	Abcam	Cat# ab110413
Mouse monoclonal anti- SIRT1	Santa Cruz	Cat# sc-74465
Rabbit monoclonal anti-SIRT2	Abcam	Cat# ab211033
Rabbit monoclonal anti-SIRT3	Cell Signaling Technology	Cat# 5490
Rabbit monoclonal anti-SOD2	Cell Signaling Technology	Cat# 13141
Rabbit monoclonal anti- FOXO3A	Cell Signaling Technology	Cat# 12829
Beta actin recombinant antibody	Proteintech	Cat# 81115-1-RR
Mouse monoclonal anti-NeuN	Abcam	Cat# ab104224
Rabbit polyclonal anti- SENP1	Novus biologicals	Cat# NB100-56405
Alexa Fluor 488 Donkey-anti-Rabbit IgG	Invitrogen	Cat# A-21206
Alexa Fluor 594 Donkey-anti-Rabbit IgG	Invitrogen	Cat# A-21207
Alexa Fluor 594 Donkey-anti-Mouse IgG	Invitrogen	Cat# A-21203
Chemicals or Commercial Assays		
N-ethylmaleimide	Sigma	Cat# 04259
DAPI ready made solution	Sigma	Cat# 28718-90-3
Pierce™ BCA Protein Assay Kits	Thermo Scientific	Cat# 23225
